# Progesterone level on the day of hCG administration in relation to the pregnancy rates of patients undergoing assisted reproduction techniques

**DOI:** 10.1590/S1679-45082017AO4091

**Published:** 2017

**Authors:** Renato de Oliveira, Fernanda Godoy Cabral, Waldemar de Almeida Pereira Carvalho, Emerson Barchi Cordts, Bianca Bianco, Caio Parente Barbosa

**Affiliations:** 1 Instituto Ideia Fértil, Faculdade de Medicina do ABC, Santo André, SP, Brazil.; 2 Pontifícia Universidade Católica de Campinas, Campinas, SP, Brazil.

**Keywords:** Pregnancy, Chorionic gonadotropin, Progesterone, Reproductive techniques, assisted, Gravidez, Gonadotropina coriônica, Progesterona, Técnicas de reprodução assistida

## Abstract

**Objective:**

To evaluate the predictive capacity for pregnancy of the progesterone level on the day of administering human chorionic gonadotropin, in women submitted to assisted reproductive techniques.

**Methods:**

An observational study with 914 women submitted to assisted reproductive techniques from August 2014 to June 2016.

**Results:**

Total pregnancy rate was 34.58%; in that, the pregnancy rate in women <35 years, between 35 and 38, and >38 years was, respectively, 42.3%, 38.7% and 16.1% (p<0.001). For embryo transfer in the same cycle, and progesterone of 1.3ng/dL, sensitivity was 4.78%, specificity, 84.18%, accuracy, 56.72%, positive likelihood ratio of 0.3019, and negative likelihood ratio of 1.1312, with receiver operating characteristic curve of 0.46 (95%CI: 0.42-0.49).

**Conclusion:**

The progesterone level on the day of administering human chorionic gonadotropin of 1.3ng/dL differs from that empirically adopted at the study site (1.7ng/dL), and has a better predictive capacity for pregnancy in the patients studied. However, the low sensitivity of this examination raises questions about its real importance.

## RESUMO

**Objetivo:**

Avaliar a capacidade preditiva para gravidez do valor de progesterona no dia da administração da gonadotrofina coriônica humana em mulheres submetidas às técnicas de reprodução assistida.

**Métodos:**

Estudo observacional com 914 mulheres submetidas a reprodução assistida de agosto de 2014 até junho de 2016.

**Resultados:**

Engravidaram 34,58% das mulheres, sendo a taxa de gravidez naquelas >35 anos, entre 35 e 38 anos e >38 anos, respectivamente, de 42,3%, 38,7% e 16,1% (p<0,001). Para o valor de progesterona até 1,3ng/dL visando à transferência embrionária no mesmo ciclo, encontraram-se sensibilidade de 4,78%, especificidade de 84,18%, acurácia de 56,72%, razão de verossimilhança positiva de 0,3019 e razão de verossimilhança negativa de 1,1312, com área sob a curva característica de operação do receptor de 0,46 (IC95%: 0,42-0,49).

**Conclusão:**

O valor de progesterona no dia da administração de gonadotrofina coriônica humana até 1,3ng/dL difere daquele empiricamente adotado no local do estudo (1,7ng/dL) e apresenta melhor capacidade preditiva para gravidez nas pacientes estudadas. No entanto, a baixa sensibilidade deste exame abre questionamentos sobre sua real importância.

## INTRODUCTION

The results of assisted reproductive techniques (ART) depend on both patient-related clinical aspects and embryo cleavage and development.^(^ ^1^ ^)^

In this process, searching for success predictors of high-complexity ART, such as *in vitro* fertilization (IVF) and intracytoplasmic sperm injection (ICSI), is key to enable single embryo transfer with acceptable pregnancy rate and reduction of multiple pregnancy-related risks.^(^ ^2^ ^)^

Several studies^(^ ^3^ ^-^ ^5^ ^)^ reported that increased serum progesterone levels in high-complexity ART, measured on the day that human chorionic gonadotropin (hCG) is administered, leads to poorer reproductive results.

This higher progesterone level, even when comparing hCG-triggered ovulation and the gonadotropin-releasing hormone agonist (GnRH), also has a negative effect on pregnancy rate,^(^ ^6^ ^)^ corroborating the importance of such evaluation.

The level of ≥1.0ng/mL is considered a premature increase in progesterone or early luteinization.^(^ ^7^ ^,^ ^8^ ^)^ This increase correlates with greater response to controlled ovarian hyperstimulation (COH), greater number of oocytes captured and higher estradiol levels (E_2_).^(^ ^9^ ^)^ The higher E_2_ level would stimulate granulosa cells to produce more progesterone, but at insufficient levels to trigger ovulation.^(^ ^10^ ^)^

Although routine use of ART provides better knowledge about cycles, early luteinization occurs in approximately 5 to 50% of cases.^(^ ^11^ ^-^ ^13^ ^)^

The increase in progesterone could have a negative effect both on ovaries in terms of oocyte maturation, fertilization and embryonic cleavage,^(^ ^12^ ^-^ ^16^ ^)^ and on endometrium, leading to inappropriate decidualization.^(^ ^17^ ^)^

In relation to values, the proposal is to consider up to 1.5ng/mL for poor responders, 1.75ng/mL for intermediate responders, and 2.25ng/mL to high responders.^(^ ^18^ ^)^ Additionally, extreme progesterone levels were associated to poor pregnancy outcome.^(^ ^19^ ^)^

Therefore, identifying the progesterone level to define embryo transfer and improve pregnancy rates justifies the importance of this investigation. In addition, there are scarce studies on highly miscegenated populations, like the Brazilian people. ^(^ ^20^ ^)^

## OBJECTIVE

To analyze progesterone level measured on measured on the day that human chorionic gonadotropin is administered, which predicts better pregnancy rates in patients undergoing assisted reproductive techniques.

## METHODS

A cross-sectional study assessing 1,200 electronic records of patients submitted to ART from August 2014 to June 2016, at the *Instituto Ideia Fértil*, and approved by the Ethics Committee of *Faculdade de Medicina do ABC* through opinion no. 676.628, CAAE: 31010214.3.0000.0082.

A total of 914 patients were selected and 286 were excluded due to incomplete records.

Progesterone was tested by Elecsys 1010 Immunoanalyzer (Roche, Indianapolis, USA) only on the day of hCG administration, since the local protocol does not measure it on the beginning of the cycle. If progesterone level was >1.7ng/mL, all formed embryos were cryopreserved for subsequent transfer.

The characteristics evaluated were age, infertility (primary or secondary), smoking, body mass index (BMI), total antral follicle count (AFC), number of follicles >14mm, number of metaphase I (MI) and II (MII) oocytes, number of formed embryos, and a variable known as “response to treatment”, defined as number of follicles >14mm, divided by total AFC multiplied by 100, to evaluate and estimated quality of COH (in percentage).

The COH protocol was defined after initial investigation using follicle-stimulating hormone receptor (FSHr) (Puregon^®^ or Gonal^®^, 100UI, 150UI or 200UI) and GnRH-antagonist (ORGALUTRAN^®^) or GnRH-agonists (both long-term and short-term protocols) taking clinical characteristics into consideration, total AFC and patient’s specific aspects, based on the local protocol. Ovulation was triggered through administration of hCG (Ovidrel^®^ *).* After 35 hours, ovarian puncture was performed and luteal phase support was initiated with vaginal micronized progesterone (600mg per day).

For statistical analysis, Groups A and B were related to pregnancy or no pregnancy after ART, respectively.

The qualitative variables were evaluated by absolute and relative frequencies using the ***χ*** ^2^ test. The quantitative variables by medians, 25% and 75% percentiles, confidence interval (CI) and Shapiro-Wilk and Mann-Whitney tests. Sensitivity and specificity were determined by the Receiver Operating Characteristic (ROC) curve, with likelihood ratio and a 95% confidence level. The statistical program used was Stata^®^ 11.0.

## RESULTS

The clinical characteristics of the groups are shown in table 1.

**Table 1:** Characterization of clinical data of evaluated patients

Clinical characteristics	Group A n (%)	Group B n (%)
Infertility of the couple		
Primary	441 (73.6)	248 (78.7)
Secondary	158 (26.4)	67 (21.3)
Past history of miscarriage		
Yes	69 (11.5)	40 (12.7)
No	530 (88.5)	275 (87.3)
Smoking		
Yes	40 (6.7)	21 (6.7)
No	559 (93.3)	294 (93.3)
BMI		
<25kg/m^2^	356 (59.4)	185 (58.7)
≥25kg/m^2^	243 (40.6)	130 (41.3)
	Median (p25-75)	
Age (years)	37 (33-40)	35 (31-38)
Infertility time (years)	3 (2-5 )	3 (2-5)
Menarche	13 (12-14)	13 (12-14)

Group A: women who did not get pregnant after assisted reproductive technique; Group B: women who got pregnant after assisted reproductive technique.

BMI: body mass index.

Table 2 displays the high-complexity treatment results. COH data included total AFC, number of follicles >14mm on the day of ovarian puncture, and treatment response. Data related to the procedure and laboratory progression results, such as MI, MII and number of embryos evolved until transfer or cryopreservation were also evaluated.

**Table 2:** Clinical laboratory parameters of assisted reproduction treatments

Laboratory data	Group A (Mean±SD)	Group B (Mean±SD)
COH data		
Total AFC	8.16±4.98	10.36±6.41
Follicle >14mm	6.11±3.85	7.2±4.32
Treatment response (%)	92.14±67.28	82.72±58.92
Laboratory data		
MI	0.44±0.94	0.61±2.01
MII	4.31±3.35	5.09±3.52
Number of embryos	1.85±1.86	3.30±2.24

Group A: Women who did not get pregnant after assisted reproduction; Group B: women who got pregnant after assisted reproduction.

SD: standard deviation; COH: controlled ovarian hyperstimulation; total AFC: total antral follicle count; MI: number of oocytes in metaphase I; MII: number of oocytes in metaphase II.

As to reproductive outcomes, total pregnancy rate was 34.6%. The evaluation of this rate considered the number of women by age group: patients under <35 years, between 35 and 38 years, and >38 years was 155 (42.3%), 126 (38.7%) and 36 (16.1%), respectively (p<0.001).

The median levels of progesterone in Groups A and B were 0.7ng/dL (95%CI: 0.65-0.71) and 0.69ng/dL (95%CI: 0.6-0.8), respectively (p=0.110).

In patients with BMI <25kg/m^2^, 356 (65.8%) from Group A had the same median progesterone (0.7ng/dL) as compared to Group B, which comprised 185 (34.2%) patients (p=0.056). Likewise, patients with BMI ≥25kg/m^2^ had the same median progesterone level (0.63ng/dL) both in Group A, with 243 (65.2%) patients, and in Group B, with 130 (34.8%) patients (p=0.407).

**Figure 1:**
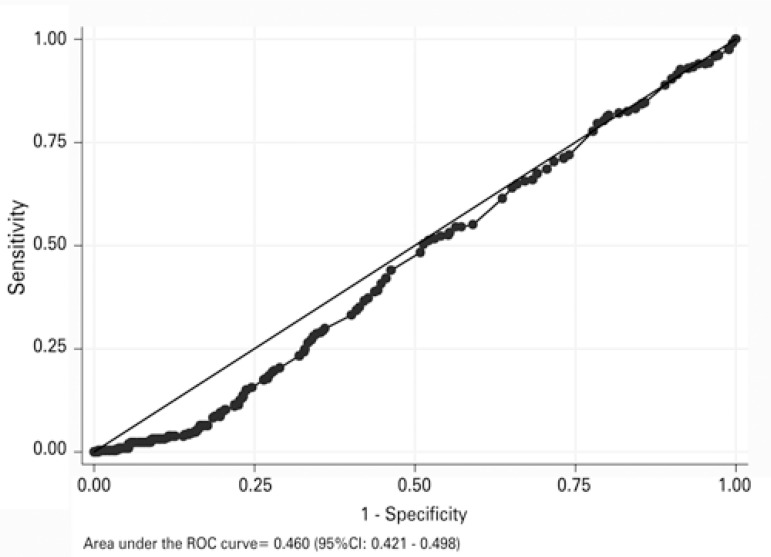
Receiver operation characteristic curve of predictive progesterone capacity in relation to pregnancy rate

The progesterone level of 1.3ng/dL obtained by ROC curve was the most representative pregnancy predictor, considering sensitivity of 4.78%, specificity of 84.18%, accuracy of 56.72%, positive likelihood ratio of 0.301, negative likelihood ratio of 1.131 and area under the curve of 0.460 (95%CI: 0.421-0.498), as shown in figure 1.

95%CI: 95% confidence interval.

## DISCUSSION

In spite of excluding approximately 84% of patients who would not get pregnant by ART, the level of 1.3ng/dL of progesterone would included only approximately 5% of those patients who got pregnant. This fact raises questions about its use.

No consensus was reached on the ideal progesterone level for embryo transfer.^(^ ^21^ ^)^ However, an analysis of more than four thousand cycles demonstrated that levels >1.5ng/mL reduced pregnancy rate.^(^ ^22^ ^)^

There have been many attempts to select groups to define the best progesterone level. For example, cycles with only GnRH antagonists and progesterone level of ≤1.5ng/mL, obtained acceptable pregnancy rate only for normal responders (6 to 18 oocytes), but not for hyper-responders (>18 oocytes).^(^ ^23^ ^)^ A systematic review and a meta-analysis with more than 60 thousand cycles concluded that the high level of progesterone decreased the pregnancy rate in fresh embryo transfer, but not frozen-thawed embryos.^(^ ^24^ ^)^

The transfer of frozen-thawed embryos is a practice already used in many centers, considering that a hyperstimulated endometrium, which is typical of COH in the same cycle, would affect embryo implantation.^(^ ^25^ ^)^ The reduced progesterone level of 1.7ng/mL adopted is expected to increase the number of embryo transfers and, therefore, improve positive outcome.

Nonetheless, the new level progesterone of 1.3ng/mL suggests some reflection about its use.

First, it is worth mentioning this level was obtained through the analysis of Brazilian patients, and this could encourage national investigation on the topic. However, there are contradictions about its adoption, as previously mentioned, since it included 5% of patients that got pregnant.

The change in the empirically adopted progesterone level from 1.7 to 1.3ng/dL and based on international populations, considering the national population studied, allows excluding most patients who would not become pregnant. Thus, at an acceptable cost, there is an attempt to prevent miscarriage with improved pregnancy rate by transfer. Moreover, search for new gestational predictors is encouraged.

The decrease in pregnancy rate with ageing is corroborated by the literature and suggests effectiveness of treatments.^(^ ^26^ ^,^ ^27^ ^)^

As limiting factors of the study, we could mention the GnRH antagonist or agonist protocols were not evaluated separately. The lack of detailed information in evaluations is a bias. The expressive number of patients, however, minimizes differences between groups.

The major benefit of this study was the readjustment of progesterone level measured on the day of hCG administration, to define embryo transfer in the same cycle at the study site. This could also provide better results in other human reproduction centers in our country.

## CONCLUSION

The progesterone level on the day of administering human chorionic gonadotropin of up to 1.3ng/dL differs from the level empirically adopted at the study center (1.7ng/dL). Although low sensitivity of this test enables arguing about its relevance, its permanence, associated with search for new pregnancy predictors, are considered essential to improve pregnancy rate per single embryo transfer.
